# Prognostic factors in tongue cancer – relative importance of demographic, clinical and histopathological factors

**DOI:** 10.1054/bjoc.2000.1323

**Published:** 2000-08-16

**Authors:** S Kantola, M Parikka, K Jokinen, K Hyrynkangs, Y Soini, O-P Alho, T Salo

**Affiliations:** Departments of Oral & Maxillofacial Surgery, Diagnosticsand Oral Medicine, Institute of Dentistry, Oulu University Hospital, University of Oulu, Aapistie 3, Oulu, 90220, Finland; Departments of Otorhinolaryngology, Pathology, Oulu University Hospital, P.O. Box 22, Oulu, 90221, Finland

**Keywords:** squamous cell carcinoma, survival, immunohistochemistry

## Abstract

The incidence of and mortality from squamous cell carcinoma (SCC) of the tongue have increased during the recent decades in the Western world. Much effort has been made to predict tumour behaviour, but we still lack specific prognostic indicators. The aim of our study was to evaluate the relative importance of the known demographic, clinical and histological factors in a homogeneous population-based group of patients with SCC of the mobile tongue. The demographic and clinical factors were reviewed retrospectively from primary and tertiary care patient files. Histological prognostic factors were determined from pre-treatment biopsies. The TNM stage was found to be the most important prognostic factor. In particular, local spread outside the tongue rather than spread to regional lymph nodes was related to poor prognosis. Several demographic and histopathological factors were closely related to TNM stage. When the cases were divided into stage I–II carcinomas and stage III–IV carcinomas, it appeared that the patient’s older age (> 65 years), a high malignancy score and an absence of overexpressed p53 protein were associated with a poorer prognosis in stage I–II carcinomas. Such cases may require more aggressive treatment. Among patients with stage III–IV carcinomas, heavy use of alcohol was significantly associated with a poor disease-specific survival time. © 2000 Cancer Research Campaign


British Journal of Cancer (2000) **83**(5), 614–619
© 2000 Cancer Research Campaign
doi: 10.1054/ bjoc.2000.1323, available online at http://www.idealibrary.com on IDEAL®

### Prognostic factors in tongue cancer – relative importance of demographic, clinical and histopathological factors

**S Kantola[1], M Parikka[2], K Jokinen[3], K Hyrynkangs[3], Y Soini[4], O-P Alho [3a] and T Salo[2,4a]**

Departments of [1]Oral & Maxillofacial Surgery and [2]Diagnostics and Oral Medicine, Institute of Dentistry, Oulu University Hospital, University of Oulu, Aapistie 3, 90220 Oulu, Finland; [3]Departments of Otorhinolaryngology and [4]Pathology, Oulu University Hospital, P.O. Box 22, 90221 Oulu, Finland

**Summary** The incidence of and mortality from squamous cell carcinoma (SCC) of the tongue have increased during the recent decades in the Western world. Much effort has been made to predict tumour behaviour, but we still lack specific prognostic indicators. The aim of our study was to evaluate the relative importance of the known demographic, clinical and histological factors in a homogeneous population-based group of patients with SCC of the mobile tongue. The demographic and clinical factors were reviewed retrospectively from primary and tertiary care patient files. Histological prognostic factors were determined from pre-treatment biopsies. The TNM stage was found to be the most important prognostic factor. In particular, local spread outside the tongue rather than spread to regional lymph nodes was related to poor prognosis. Several demographic and histopathological factors were closely related to TNM stage. When the cases were divided into stage I–II carcinomas and stage III–IV carcinomas, it appeared that the patient's older age (> 65 years), a high malignancy score and an absence of overexpressed p53 protein were associated with a poorer prognosis in stage I–II carcinomas. Such cases may require more aggressive treatment. Among patients with stage III–IV carcinomas, heavy use of alcohol was significantly associated with a poor disease-specific survival time. © 2000 Cancer Research Campaign

**Keywords:** squamous cell carcinoma; survival; immunohistochemistry

The incidence of oral squamous cell carcinoma (SCC) has increased over the past decades in Europe and in the United States. At the same time, mortality from the disease has increased as well (Depue, 1986; Davis and Severson, 1987; Macfarlane et al, 1996). The management of oral SCC is notably dependent on the TNM staging system, which is based on the clinical evaluation. The stage, however, is not always sufficient for prognostication. For example, some small T1 tumours behave in an aggressive manner and have an unexpectedly poor prognosis. Thus, it would be of great benefit to be able to identify the more aggressive tumours at the time of diagnosis. There are numerous previous publications dealing with the identification of demographic, clinical and histological prognostic factors (Janot et al, 1996; Gluckman et al, 1997). However, apart from TNM stage, there is controversy over the relative importance of different prognostic factors.

Since the TNM stage has been found to be the most important prognostic factor for oral SCC, there are studies of specifically TNM stage I tumours (Högmo et al, 1999) or T1–2 tumours (Leedy et al, 1994), for example. Nevertheless, to our knowledge, there are no previous studies in which the prognostic factors would have been compared in all stages. The aim of the present paper was to assess the relative importance of the known prognostic demographic, clinical and histological factors in a population-based sample of 105 patients with SCC of the mobile tongue. Furthermore, we investigated whether distinctive prognostic factors could be found for stage I–II and for stage III–IV carcinomas.

*Received 13 October 1999*
*Revised 8 May 2000*
*Accepted 8 May 2000*

*Correspondence to:* S Kantola

### PATIENTS AND METHODS

### Patients

The study area comprised the two northernmost provinces of Finland with a population of approximately 700 000. The Oulu University Hospital is the only tertiary centre in the area, and all the patients with tongue cancer are treated there. All the patients who lived in the area and were diagnosed with cancer of the tongue (International Classification of Diseases, version 9, code 141) between January 1st 1974 and December 31st 1994 were manually identified from the surgical, radiotherapy and discharge registers of the Oulu University Hospital. Carcinomas extending over various anatomical structures were included, whenever the tongue was clinically and without a doubt detected to be the site of origin. Tumours of the base of the tongue (International Classification of Diseases, version 9, code 1410) were excluded from the study. Altogether 108 new cases of tongue cancer were detected during the period 1974–1994. Three cases were excluded because of insufficient clinical data.

### Demographic factors

Data concerning the following demographic factors were gathered from the clinical records: sex, age at the time of diagnosis (44 years or under, 45–65 years and over 65 years), socio-economic

[a]Last two authors have equally contributed to the work.

status (United Nations, 1978), place of residence (urban vs. rural) (Statistics Finland, 1993), smoking (current or former smoker vs. non-smoker) and alcohol consumption (none, light, moderate vs. heavy). Alcohol consumption was defined as heavy if the patient drank daily or he/she had been diagnosed as an alcoholic.

### Clinical factors

The following clinical factors were gathered from the patient's primary care or hospital files: clinical appearance of the carcinoma (exophytic vs. indurated, ulcerative, crater), carcinoma spread outside the tongue (yes vs. no), neck nodes at the time of diagnosis (yes vs. no), and the TNM stage classification of the tumour (International Union Against Cancer, 1987).

### Histopathological factors

All the histological factors were determined from paraffin sections of pre-treatment biopsies. Eight cases were excluded because of poor specimen quality. During the study, unfortunately, several paraffin blocks were exhausted, and only 55 samples were available for analysis of apoptosis. The histological sections (with the exception of WHO grading) were analysed by two of the authors suitably trained in scoring. The determination was done in a blinded manner without a knowledge of the patient outcome. The scores were based on consensus between the two authors. The following factors were determined from the sections.

Tumour grade according to the WHO classification (well differentiated, moderately differentiated or poorly differentiated, World Health Organization, 1997), as given by the pathologist, was retrospectively reviewed from the patients' hospital files. The malignancy scores of the carcinomas were determined using the method introduced by Bryne et al (1992). In this method, the score is determined from the deep invasive tumour margins. We determined the score from the most lateral invasive margin of the tumour present on the sample, as the deep invasive margin of the tumour was only present in 26 (29%) of the biopsy samples. A score from 1 to 4 is given for five morphological features: degree of keratinization, nuclear polymorphism, number of mitoses, pattern of invasion and lymphoplasmacytic infiltration. The subscores are then summed up into a total malignancy score (5–20). In the analysis, the cases were divided into three groups based on the malignancy score: 5–10 points, 11–15 points and 16–20 points.

Tumour angiogenesis was determined from sections immunohistochemically stained against factor VIII (Dako, A/S, Glostrup, Denmark), the method used being similar to that of Leedy et al (1994). The microvessels, excluding single cells, were counted in ten high-power fields within the tumour (objective $\times$ 40; diameter of the field 400 μm). The mean value of the vessels in the ten fields was counted, and the results were divided into three groups based on the quartiles of the means for the analysis of tumour angiogenesis.

Inflammatory cells (polymorphonuclear, neutrophilic and eosinophilic leucocytes, lymphocytes and plasma cells) were counted on the tumour margin (objective $\times$ 40, diameter of the field 400 μm). The total extent of the inflammatory infiltrate was categorised as light (+), moderate (++) or heavy (+++).

Nerve invasion was determined by using S-100-stained sections (Dako, A/S, Glostrup, Denmark). Nerve invasion was considered positive if there were clearly recognisable SCC cells in any of the nerves present in the biopsy section.

To determine tumour apoptosis, 3′ -end labelling of apoptotic cell DNA was performed using the ApopTag® in situ apoptosis detection kit (Oncor, Gaithersburg, MD) and following the manufacturer's instructions with a few modifications as described by Soini and Pääkkö (1996). Apoptotic cells were counted using a similar method as applied to microvessels. For the analysis of apoptosis, the results were divided into three groups based on the quartiles of the means.

The presence of human papilloma virus DNA (HPV 6, 11, 16, 18, 31 and 33 subtypes) in the sections was ascertained by in situ hybridization as described by Soini et al (1996). Human papilloma virus was also detected by PCR, using the HPV consensus primers MY 09 and MY 11 (Soini et al, 1996), β-actin by 5′ sense and 3′ antisense primers according to Ponte et al (1984).

Overexpression of p53 protein was determined from sections using similar method as described by Soini and Pääkkö (1996). Immunohistochemical staining was done using the polyclonal rabbit anti-human p53 antibody CM1 diluted 1:1000 and the secondary anti-rabbit antibody as well as the avidin-biotin peroxidase complex (Vectastain® ABC kit, Vector laboratories, Burlingame, CA). The expression of p53 was recorded as negative (–), weakly positive (+) or strongly positive (++).

### Treatment of patients

Small T1N0M0 carcinomas were treated with local radical surgery without neck dissection. In the case of T2N0M0 carcinomas, modified upper neck dissection was included in the treatment. Larger and more widely spread carcinomas were treated with a combination of hemiglossectomy and radical neck dissection and adjacent postoperative radiotherapy. The most advanced, inoperable cases were treated with radiotherapy only. Sixty-six (63%) of the patients had surgery, 20 (19%) radiotherapy and 11 (10%) both surgery and adjuvant radiotherapy. Five (5%) of the patients refused surgery and were thus treated with radiotherapy alone, and three (3%) patients did not receive any treatment.

### Statistical methods

The inter-relationships between the various prognostic factors were assessed with the chi-square method. The overall and disease-specific mean survival and recurrence-free times and their 95% confidence intervals (95% CI) were calculated according to the Kaplan-Meier method. Furthermore, disease-specific 75th percentile survival times and their ranges were calculated and compared with Breslow's test. The disease-specific survival time was determined from the date the patient first received treatment for the cancer. The tumour status of each patient was recorded based on the data of the latest control visit at the referral centre. The prognostic effects of demographic and histopathological factors were studied separately in stage I–II and stage III–IV carcinomas. In the analyses, $P$ values ≤0.05 were considered statistically significant.

### RESULTS

### Overall prognosis

The mean overall survival time was 99 months (95% CI 80–118) and the disease-specific survival time 145 months (95%

*© 2000 Cancer Research Campaign* 

**Table 1** The disease-specific 75th percentile survival times (months) and range for patient-related prognostic factors in a series of 105 patients with cancer of the tongue

| Factors (*n*) | 75th percentile survival time | Range | *P* |
|---|---|---|---|
| Gender | | | 0.94 |
| Female (60) | 19 | (2–43) | |
| Male (45) | 17 | (3–91) | |
| Age group | | | 0.02 |
| 44 yrs and under (12) | >60[b] | | |
| 45–65 yrs (48) | 22 | (3–91) | |
| over 65 yrs (45) | 13 | (2–43) | |
| Socio-economic status[a] | | | 0.31 |
| Low (71) | 19 | (2–91) | |
| High (21) | >60[b] | | |
| Domicile | | | 0.66 |
| Urban (68) | 19 | (3–91) | |
| Rural (37) | 17 | (2–32) | |
| Smoking* | | | 0.49 |
| No (38) | 91 | (6–91) | |
| Yes (49) | 32 | (5–32) | |
| Alcohol consumption[a] | | | 0.05 |
| None, seldom, moderate (54) | >60[b] | | |
| Heavy (15) | 12 | (3–17) | |

[a]Calculated for those with data available. [b]The disease-specific survival rate was over 75%

CI 125–166). The 75th percentile disease-specific survival time was 19 months (range 2, 91). A total of 42 (40%) patients developed a recurrence during the follow-up. The mean recurrence-free rate was 126 months (95% CI 105–148). The recurrences were local in 47% of the cases, in the neck in 35% and both local and in the neck in 18%.

### Distributions and prognostic effects of demographic, clinical and histopathological factors in univariate analyses

Sixty (55%) of the patients were female (Table 1). The median age of the patients was 64 years (range 26–90). Seventy-seven percent of the patients had a low socio-economic status and 65% lived in an urban domicile. Over half of the patients were smokers and 22% were heavy alcohol users. The patient's older age and heavy use of alcohol decreased significantly the disease-specific survival time (*P* values 0.02 and 0.05, respectively). None of the other demographic factors had a significant effect on survival (Table 1).

There were 44 (44%) stage I–II carcinomas in the study, and in forty-three (41%) cases the carcinoma had spread to the neck nodes by the time of diagnosis (Table 2). The median size of the carcinomas was 30 m. The TNM stage had the most significant effect on the patient's mean disease-specific survival time ($P$ = 0.0001) (Table 2). Particularly local spread outside the tongue had a significant effect on prognosis ($P$ = 0.04). Tumour size, the presence of positive neck nodes at the time of diagnosis or the clinical appearance of the carcinoma did not have a statistically significant effect on disease-specific survival (Table 2).

Three (3%) of the patients had a poorly differentiated neoplasm (Table 3). Only five (6%) of the cases had a high (16–20 points) malignancy score. None of the histopathological factors had a statistically significant effect on disease-specific survival (Table 3). Nevertheless, a low malignancy score and a heavy tumour angiogenesis (mean of 8.5 vessels or over) tended to have a positive influence on survival time. Also, when there was a heavy inflammatory response at the tumour edge, the disease-specific survival time tended to be longer. We were unable to detect human papilloma virus in any of the biopsies analysed.

**Table 2** The disease-specific 75th percentile survival times (months) and range for clinical prognostic factors in a series of 105 patients with cancer of the tongue

| Factors (*n*) | 75th percentile survival time | Range | *P* |
|---|---|---|---|
| Clinical stage[a] | | | 0.0001 |
| I (14) | 36 | (14–91) | |
| II (30) | >60[b] | | |
| III (41) | 19 | (8–43) | |
| IV (16) | 5 | (3–32) | |
| Carcinoma spread outside the tongue | | | 0.04 |
| No (82) | 31 | (2–91) | |
| Yes (23) | 12 | (3–32) | |
| Size of the carcinoma[a] | | | 0.38 |
| 20 mm or under (20) | 91 | (8–91) | |
| 21–40 mm (62) | 17 | (2–32) | |
| over 40 mm (20) | 12 | (4–22) | |
| Neck nodes at the time of diagnosis[a] | | | 0.25 |
| No (61) | 31 | (2–91) | |
| Yes (43) | 14 | (3–32) | |
| Appearance of the carcinoma[a] | | | 0.93 |
| Exophytic (32) | >60[b] | | |
| Ulcerative/crater (34) | 22 | (9–32) | |

[a]Calculated for those with data available. [b]The disease-specific survival rate was over 75%

**Table 3** The disease-specific 75th percentile survival times (months) and range for histopathological prognostic factors in a series of 105 patients with cancer of the tongue

| Factors (*n*) | 75th percentile survival time | Range | *P* |
|---|---|---|---|
| Grade | | | 0.59 |
| Well differentiated (44) | 29 | (2–36) | |
| Moderately differentiated (40) | >60[a] | | |
| Poorly differentiated (3) | 16 | (16–17) | |
| Malignancy score | | | 0.44 |
| 5–10 (38) | 43 | (6–43) | |
| 11–15 (47) | 22 | (3–32) | |
| 16–20 (5) | 14 | (14–16) | |
| Tumour angiogenesis | | | 0.27 |
| 4.7 vessels and under (17) | 17 | (8–22) | |
| 4.8–8.4 vessels (31) | 31 | (6–91) | |
| 8.5 vessels and over (18) | >60[a] | | |
| Inflammatory response | | | 0.61 |
| Light (12) | 12 | (8–14) | |
| Moderate (47) | 22 | (4–91) | |
| Heavy (38) | >60[a] | | |
| Nerve invasion | | | 0.35 |
| No (49) | 29 | (5–43) | |
| Yes (7) | >60[a] | | |
| Apoptosis | | | 0.90 |
| 0.6 and under (14) | 16 | (8–16) | |
| 0.7–1.3 (25) | 91 | (4–91) | |
| 1.4 and over (16) | 22 | (3–22) | |
| Overexpression of p53 protein | | | 0.90 |
| – (37) | 19 | (6–32) | |
| + (26) | 22 | (4–91) | |
| ++ (22) | 36 | (3–36) | |

[a]The disease-specific survival rate was over 75%.

*© 2000 Cancer Research Campaign*

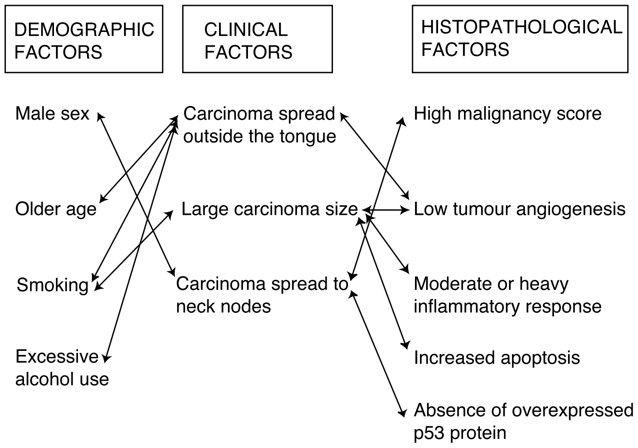

**Figure 1** Statistically significant ($P \leq 0.05$, chi-square analysis) interactions between demographic, clinical and histopathological factors marked with arrows in a series of 105 patients with cancer of the tongue

### Interaction between demographic, clinical and histopathological factors

As shown in Figure 1, several demographic and histopathological factors were significantly related to the clinical stage at the time of diagnosis. We found that male sex, older age, smoking and heavy use of alcohol correlated significantly with the stage. Furthermore, it appeared that a low microvessel density, a high malignancy score of the carcinoma, a heavy inflammatory response at the edge of the carcinoma, increased apoptosis and an absence of overexpressed p53 protein correlated significantly with the clinical distribution of the carcinoma. In contrast, there were no statistically significant correlations between any of the demographic and histopathologic factors studied.

### Prognostic factors in stage I–II carcinomas

Patient's age over 65 years, a high malignancy score (16–20 points) and an absence of the overexpressed p53 protein significantly shortened the mean disease-specific survival in stage I–II carcinomas (Table 4). The findings were similar, although not statistically significant, when the prognostic factors for recurrence free times were determined (data not shown).

**Table 4** The disease-specific 75th percentile survival times (ST) in months and range according to patient-related and histopathologic prognostic factors in stage I–II carcinomas and in stage III–IV carcinomas in 101 patients with cancer of the tongue (stage not available for four patients)

| | Stage I–II carcinomas | | | Stage III–IV carcinomas | | |
|---|---|---|---|---|---|---|
| | (*n*) | ST (range) | *P* | (*n*) | ST (range) | *P* |
| All patients[a] | (44) | 91 (6–91) | | (57) | 14 (3–43) | |
| Gender | | | 0.79 | | | 0.70 |
| Female | (27) | >60[b] | | (30) | 19 (5–43) | |
| Male | (17) | 31 (6–91) | | (27) | 12 (3–17) | |
| Age group | | | 0.03 | | | 0.62 |
| 65 yrs and under | (24) | >60[b] | | (32) | 14 (3–22) | |
| over 65 yrs | (20) | 29 (6–36) | | (25) | 13 (5–43) | |
| Smoking | | | 0.23 | | | 0.09 |
| No | (21) | 36 (6–91) | | (17) | >60[b] | |
| Yes | (20) | >60[b] | | (28) | 16 (5–32) | |
| Alcohol consumption | | | 0.27 | | | 0.003 |
| None–moderate | (30) | 91 (6–91) | | (23) | >60[b] | |
| Heavy | (5) | >60[b] | | (10) | 12 (3–17) | |
| Grade | | | 0.87 | | | 0.49 |
| Well-differentiated | (24) | 36 (6–36) | | (19) | 19 (5–22) | |
| Moderately diff. | (16) | >60[b] | | (21) | >60[b] | |
| Poorly differentiated | | | | (3) | 16 (16–17) | |
| Malignancy score | | | 0.05 | | | 0.84 |
| 5–10 | (20) | >60[b] | | (18) | 43 (8–43) | |
| 11–15 | (17) | >60[b] | | (27) | 17 (3–32) | |
| 16–20 | (3) | 14 (14–16) | | (2) | >60[b] | |
| Angiogenesis | | | 0.16 | | | 0.09 |
| Under 8.5 vessels | (24) | 31 (6–91) | | (22) | 17 (9–22) | |
| 8.5 vessels and over | (7) | >60[b] | | (10) | >60[b] | |
| Inflammatory response | | | 0.32 | | | 0.07 |
| Light | (3) | >60[b] | | (7) | 9 (8–14) | |
| Moderate or heavy | (40) | 91 (6–91) | | (43) | 22 (3–43) | |
| Apoptosis | | | 0.24 | | | 0.95 |
| Under 1.4 | (16) | 91 (16–91) | | (21) | 17 (5–17) | |
| 1.4 and over | (6) | 17 (6–17) | | (9) | 22 (3–22) | |
| Overexpression of p53 protein | | 0.05 | | | | 0.49 |
| – | (16) | 17 (6–29) | | (21) | 19 (9–32) | |
| +–++ | (21) | >60[b] | | (25) | 17 (3–43) | |

[a]*P* for the difference between stage I–II and stage III–IV carcinomas was 0.01. [b]The disease-specific survival rate was over 75%

© 2000 Cancer Research Campaign 

### Prognostic factors in stage III–IV carcinomas

Heavy use of alcohol was significantly related to a poorer prognosis in stage III–IV carcinomas (Table 4). Furthermore, smoking, low or moderate tumour angiogenesis and a slight inflammatory response at the edge of the carcinoma tended to correlate with a poor prognosis, even though the correlation was not significant. Again, similar findings were achieved when the prognostic factors for recurrence-free times were determined (data not shown).

### DISCUSSION

We analysed the relative importance of demographic, clinical and histopathological factors in a population-based study of 105 tongue SCC patients. SCC of the tongue is the most common intraoral malignancy and is usually detected at a relatively early stage. In the present patient population, however, there were 57 (56%) patients with stage III–IV carcinomas. The prognosis of tongue cancer is better than the prognosis of head and neck SCCs in general. The prognostic importance of the TNM stage was once again confirmed by the present findings. It was clearly shown that the patients with TNM Stage IV carcinomas had a significantly poorer prognosis than the patients with smaller and more localized tumours. One must remember, however, that in the present patient material the treatment was planned according to the TNM stage. The result confirms the findings of several previous studies (Ghouri et al, 1993; Bundgaard et al, 1996; Janot et al, 1996). Particularly local spread outside the tongue rather than spread to regional lymph nodes was significantly related to a poor prognosis.

It was noted that several demographic and histopathological factors were significantly related to the clinical distribution of the carcinoma. From a clinical point of view, it is of great interest to find out the most important prognostic factors in stage I–II cases. Patient's older age, a high Bryne's malignancy score and an absence of overexpressed p53 protein predicted poorer prognosis in stage I–II carcinomas. In the case of stage III–IV carcinomas, heavy use of alcohol was significantly associated with a short survival time. Since the overall survival of the disease was good, 63%, median survival times could not be calculated. Instead, we used 75th percentile survival times and ranges, which were more appropriate here.

The patient's older age significantly shortened the disease-specific survival time in the present study. The result is in accordance with a previous study, where head and neck SCC patients aged over 60 years had a significantly poorer prognosis than younger patients (Janot et al, 1996). However, contradictory data also exist. Friedlander et al (1997) noted that the patient's younger age was associated with aggressive tumour behaviour in tongue cancers.

The prognostic value of Bryne's malignancy score in oral cancers has been shown earlier by other authors (Piffkò et al, 1997). The present study is the first to our knowledge to establish the prognostic significance of the malignancy score, specifically in stage I–II tongue cancers. There were, however, only three stage I–II carcinomas in the group with the highest malignancy scores. This might reflect the less aggressive nature of tongue cancer compared to the majority of head and neck cancers. In a study by Högmo et al (1999) of stage I tongue cancer patients no correlation between the malignancy score and regional recurrence of the disease was found. In that particular study, however, Bryne's malignancy score was analysed factor by factor and not as a total score. It is thus impossible to conclude whether the score itself would have had a prognostic value.

According to the present study it seems that an absence of the overexpressed p53 leads to a poorer prognosis in stage I–II carcinomas. The finding is quite the opposite compared to a recent Swedish study, where the presence of p53 predicted regional recurrence in early stage tongue carcinomas (Högmo et al, 1999). According to Leedy et al (1994), however, p53 did not have any prognostic value in T1 and T2 tongue carcinomas. Similarly, the study by Gluckman et al (1997) revealed no association between the presence of p53 and tumour aggressiveness. In the present study the overexpression of p53 protein was determined with immunohistochemical (IHC) staining. False negative results may occur when the PCR method is used, as it tests only certain exons (5–8) of the protein, leaving the mutations possibly occurring in other exons undetected (Soussi et al, 1994). Saunders et al (1999) performed full-length gene sequencing and IHC staining to determine p53 in laryngeal carcinomas. Nevertheless, they did not find a correlation between the presence of p53 and the recurrence rate of carcinomas.

There was a trend to suggest that a heavy angiogenesis is related to a good prognosis in stage III–IV carcinomas, contradicting the earlier findings (Gasparini et al, 1993; Shpitzer et al, 1996), but agreeing with the study by Janot et al (1996), where head and neck SCC patients with higher microvessel counts had better two-year overall survival rates and lower rates of locoregional failures and metastasis. The number of microvessels was small in the present study compared to the previous studies (Leedy et al, 1994; Gluckman et al, 1997). This was most probably due to the fact that the microvessels were counted within the carcinoma and not in the 'hot spot' area, nor were single stained cells considered microvessels. None of the staining methods presently in use (factor VIIIAg, CD-31 and CD-34) are able to distinguish between neovascularization and the vessels that existed before tumour development. Moreover, it has been suggested that due to the abundant vascularity of the tongue and the oral cavity, carcinomas of this region might be less dependent on neovascularization for growth and metastasis (Gleich et al, 1997).

Human papillomavirus (HPV) was not found in a single case in this patient population. This was quite surprising in view of how much published data are available concerning the correlation between the expression of HPV and oral SCC. However, there are also some previous studies with similar results (Zeuss et al, 1991; Matzow et al, 1998). In a recent study by Pintos et al (1999) on upper aerodigestive tract neoplasms, HPV was detected in three out of 26 oral SCCs. The authors noted that the HPV detection rate was higher for pharyngeal carcinomas than for tumours of the mouth or the larynx. We used two sensitive methods to detect HPV, PCR and in situ hybridization, which makes our results reliable. In the analysis PCR by β-actin primers gave a positive result in every sample. This indicates that the amplification of genomic DNA was successful and false negative results were excluded. Furthermore, the detection of HPV was done with exactly similar techniques as in the study by Soini et al (1996), where HPV DNA was found in 30% of the lung carcinoma samples studied.

As a conclusion, according to the present study, the TNM stage and particularly the local spread of the carcinoma at the time of diagnosis significantly correlated with the disease-specific

© 2000 Cancer Research Campaign survival time in patients with SCC of the tongue. In stage I–II carcinomas the patient's older age (> 65 years), a high malignancy score and an absence of overexpressed p53 protein had a profound effect on disease-specific survival. According to this study, small, localized carcinomas in such cases may require more aggressive treatment.

### REFERENCES

Bryne M, Koppang HS, Lilleng R and Kjærheim Å (1992) Malignancy grading of the deep invasive margins of oral squamous cell carcinomas has high prognostic value. *J Pathol* **166**: 375–381

Bundgaard T, Benzen SM, Wildt J, Sørensen FB, Søgaard H and Nielsen JE (1996) Histopathologic, stereologic, epidemiologic, and clinical parameters in the prognostic evaluation of squamous cell carcinoma of the oral cavity. *Head Neck* **18**: 142–152

Davis S and Severson RK (1987) Increasing incidence of cancer of the tongue in the United States among young adults. *Lancet* **2**(8564): 910–911

Depue RH (1986) Rising mortality from cancer of the tongue in young white males. *N Engl J Med* **315**: 647

Friedlander PL, Schantz SP, Shaha AR, Yu G and Shah JP (1997) Squamous cell carcinoma of the tongue in young patients: a matched-paired analysis. *Head Neck* **20**: 363–368

Gasparini G, Weidner N, Maluta S, Pozza F, Boracchi P, Mezzetti M, Testolin A and Bevilacqua P (1993) Intratumoral microvessel density and p53 protein: correlation with metastasis in head and neck squamous-cell carcinoma. *Int J Cancer* **55**: 739–744

Ghouri AF, Zamora RL, Harvey JE, Spitznagel Jr EL, Sessions DG (1993) Epidermoid carcinoma of the oral cavity and oropharynx: validity of the current AJCC staging system and new statistical tools for the prediction of subclinical neck disease. *Otolaryngol Head Neck Surg* **108**(3): 225–232

Gleich LL, Biddinger PW, Duperier FD and Gluckman JL (1997) Tumor angiogenesis as a prognostic indicator in T2–T4 oral cavity squamous cell carcinoma: a clinicopathologic correlation. *Head Neck* **19**: 276–280

Gluckman JL, Pavelic ZP, Welkoborsky H-J, Mann W, Stambrook P, Gleich L, Wilson K, Righi P, Portugal LG, McDonald J, Biddinger P, Steward D and Gartside P (1997) Prognostic indicators for squamous cell carcinoma of the oral cavity: A clinicopathologic correlation. *Laryngoscope* **107**: 1239–1244

Högmo A, Kuylentierna R, Lindholm J and Munck-Wikland E (1999) Predictive value of malignancy grading systems, DNA content, p53, and angiogenesis for stage I tongue carcinomas. *J Clin Pathol* **52**: 35–40

International Union Against Cancer (1987) *TNM classification of malignant tumours* 4th edn. Springer-Verlag: Berlin

Janot F, Klijanienko J, Russo A, Mamet J-P, de Braud F, EI-Naggar AK, Pignon J-P, Luboinski B and Cvikovic E (1996) Prognostic value of clinicopathological parameters in head and neck squamous cell carcinoma: a prospective analysis. *Br J Cancer* **73**: 531–538

Leedy DA, Trune DR, Kronz JD, Weidner N and Cohen JI (1994) Tumor angiogenesis, the p53 antigen, and cervical metastasis in squamous cell carcinoma of the tongue. *Otolaryngol Head Neck Surg* **111**: 417–422

Macfarlane GJ, Sharp L, Porter S and Franceschi S (1996) Trends in survival from cancers of the oral cavity and pharynx in Scotland: a clue as to why the disease is becoming more common? *Br J Cancer* **73**: 805–808

Matzow T, Boysen M, Kalantari M, Johansson B and Hagmar B (1998) Low detection rate of HPV in oral and laryngeal carcinomas. *Acta Oncol* **37**: 73–76

Piffkò J, Bànkfalvi À, Öfiner D, Bryne M, Rasch D, Joos U, Böcker W and Schmid KW (1997) Prognostic value of histobiological factors (malignancy grading and AgNOR content) assessed at the invasive tumour front of oral squamous cell carcinomas. *Br J Cancer* **75**: 1543–1546

Pintos J, Franco EL, Black MJ, Bergeron J and Arella M (1999) Human papillomavirus and prognoses of patients with cancers of the upper aerodigestive tract. *Cancer* **85**: 1903–1909

Ponte P, Ng SY, Engel J, Gunning P and Kedes L (1984) Evolutionary conservation in the untranslated regions of actin mRNAs: DNA sequence of a human beta-actin cDNA. *Nucleic Acids Res* **12**: 1687–1696

Saunders ME, MacKenzie R, Shipman R, Fransen E, Gilbert R and Jordan CK (1999) Patterns of p53 gene mutations in head and neck cancer: full-length gene sequencing and results of primary radiotherapy. *Clin Cancer Res* **5**: 2455–2463

Shpitzer T, Chaimoff M, Gal R, Stern Y, Feinmesser R and Segal K (1996) Tumor angiogenesis as a prognostic factor in early oral tongue cancer. *Arch Otolaryngol Head Neck Surg* **122**: 865–868

Soini Y and Pääkkö P (1996) Extent of apoptosis in relation to p53 and bcl–2 expression in germ cell tumours. *Hum Pathol* **27**: 1221–1226

Soini Y, Nuorva K, Kamel D, Pöllänen R, Vähäkangas K, Lehto V-P, Pääkkö P (1996) Presence of human papilloma virus DNA and abnormal p53 protein accumulation in lung carcinoma. *Thorax* **51**: 887–893

Soussi T, Legros Y, Lubkin R, Ory K and Schlichtolz B (1994) Multifactorial analysis of p53 alteration in human cancer: a review. *Int J Cancer* **57**: 1–9

Statistics Finland (1993) *Communities 1993*. Helsinki

United Nations (1978) *Recommendations for the 1980 censuses of population and housing: the ECE region*. In: Statistical standards and studies. No. 31: New York

World Health Organization (1997) *Histological typing of cancer and precancer of the oral mucosa* (Second Edition). Pindborg J, Reichart P, Smith C and van der Waal I (eds), Springer-Verlag, Berlin Heidelberg

Zeuss MS, Miller CS and White DK (1991) In situ hybridization analysis of human papillomavirus DNA in oral mucosal lesions. *Oral Surg Oral Med Oral Pathol* **71**: 714–720

*© 2000 Cancer Research Campaign*
